# A CRISPR platform for targeted in vivo screens identifies *Toxoplasma gondii* virulence factors in mice

**DOI:** 10.1038/s41467-019-11855-w

**Published:** 2019-09-03

**Authors:** Joanna Young, Caia Dominicus, Jeanette Wagener, Simon Butterworth, Xingda Ye, Gavin Kelly, Merav Ordan, Becky Saunders, Rachael Instrell, Michael Howell, Aengus Stewart, Moritz Treeck

**Affiliations:** 1 Signalling in Apicomplexan Parasites Laboratory, The Francis Crick Institute, 1 Midland Road, NW1 1AT London, UK; 2 Bioinformatics and Biostatistics Science Technology Platform, The Francis Crick Institute, 1 Midland Road, NW1 1AT London, UK; 3 High Throughput Screening Science Technology Platform, The Francis Crick Institute, 1 Midland Road, NW1 1AT London, UK

**Keywords:** Parasite genetics, Parasite host response, Parasite immune evasion

## Abstract

Genome-wide CRISPR screening is a powerful tool to identify genes required under selective conditions. However, the inherent scale of genome-wide libraries can limit their application in experimental settings where cell numbers are restricted, such as in vivo infections or single cell analysis. The use of small scale CRISPR libraries targeting gene subsets circumvents this problem. Here we develop a method for rapid generation of custom guide RNA (gRNA) libraries using arrayed single-stranded oligonucleotides for reproducible pooled cloning of CRISPR/Cas9 libraries. We use this system to generate mutant pools of different sizes in the protozoan parasite *Toxoplasma gondi* and describe optimised analysis methods for small scale libraries. An in vivo genetic screen in the murine host identifies novel and known virulence factors and we confirm results using cloned knock-out parasites. Our study also reveals a potential trans-rescue of individual knock-out parasites in pools of mutants compared to homogenous knock-out lines of the key virulence factor MYR1.

## Abstract (web summary)

Targeted CRISPR libraries expand the use of genetic screens across experimental conditions. Here, the authors develop a method for generating and analysing small scale custom CRISPR libraries and use it in the human and livestock pathogen *Toxoplasma gondii* to identify virulence factors in mice.

## Introduction

Genetic screens provide significant advances in our understanding of genes required for growth under variable conditions. The advent of Clustered Randomly Interspersed Short Palindromic Repeats (CRISPR) technology has supplanted previous gold standard screening methods, and has enabled the generation of large mutant pools covering the entire genome of both eukaryotic and prokaryotic organisms^1–3^. In addition to identifying genes required for fitness under standard growth conditions, genetic screens also facilitate the identification of genes that play a key role under restrictive conditions, such as under drug pressure or those involved in host–pathogen interactions. For example, genetic screens have provided insights into the host factors required for bacterial infection (e.g. enterohaemorrhagic *Escherichia coli* and *Vibrio parahaemolyticus*)^4,5^, toxin cytotoxicity^6^ and viral invasion^7^. In the parasite *Toxoplasma gondii*, a genome-wide genetic screen has been used to identify genes that are required for growth in vitro^8^. However, CRISPR screens have not yet been employed, to the best of our  knowledge, to identify virulence factors required during infection of a mammalian host.

*Toxoplasma* infects virtually any warm-blooded animal, including 1/3rd of the human population^9^. Three major *Toxoplasma* clonal lineages have been identified: type I parasites have a lethal dose (LD) of 1 parasite in laboratory mice (LD_100_ = 1), while type II and type III strains have a LD_50_ of 1000 and >10E5, respectively^10^. The differences in virulence between strains are caused by variations in the expression levels or sequences of parasite virulence factors that are secreted into the host cell. While several virulence factors have been identified by genetic crosses between parasite strains^11–13^, a substantial proportion of secreted effectors are likely to be required across all strains and require a different screening approach. Furthermore, some effectors that are essential for virulence in vivo show no growth defect in cell culture (e.g. GRA2^14^), highlighting the importance of in vivo screening for the identification of novel factors.

While genome-wide libraries have proven extremely informative, certain experimental conditions preclude their use. For instance, target cells may be particularly precious and therefore cannot be obtained in sufficient numbers to ensure adequate coverage. Alternatively, the use of large mutant pools may not be suitable given the nature of the experimental readout such as in single-cell analyses or, as shown here, in in vivo studies where the LD of a pathogen dictates the time to select for phenotypes before overwhelming the host. More specifically, the dose needs to be sufficiently low to allow parasite replication without killing the mouse, while maintaining coverage of the library. In such instances, tailored CRISPR libraries that target a subset of genes provide a powerful tool. While some previous studies have used tailored libraries^7^, these have been fixed and lacked the option to modify target genes and controls for each experiment.

Here we have established a method to rapidly generate tailored CRISPR libraries of varying scale and composition by generating an arrayed gRNA library for single-step cloning of gRNA pools into a custom CRISPR/Cas9 vector. We demonstrate reproducible knockout (KO) efficiency in *Toxoplasma *using libraries containing 200 to 3200 guides, and establish analysis methods tailored for small library sizes. Lastly, we demonstrate high reproducibility of CRISPR screens in vivo, and identify known and novel *Toxoplasma *virulence factors. Surprisingly, we observed that some gene KOs for virulence factors behave differently when present in a mutant pool than they do as homogenous clones in murine infections. The methodology established here provides a versatile and inexpensive platform for CRISPR library generation that is widely applicable.

## Results

### Modified pCas9 plasmid shows robust KO of target genes

**Fig. 1:**
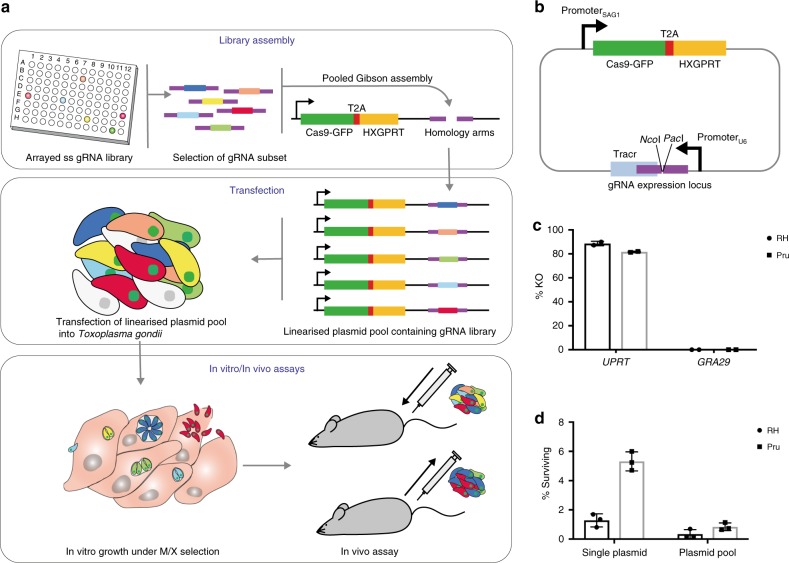
Single-step CRISPR knockout pools from arrayed single-stranded oligos. **a** Schematic of tailored CRISPR screening method. gRNA encoding oligonucleotides are arrayed in multiwell plates and the gRNA of interest are selected for pooled Gibson cloning into pCAS9-T2A-HXGPRT. The vector library is transfected into parasites to generate a pool of mutants for testing in vitro and in vivo. **b** Schematic of pCAS9-T2A-HXGPRT. Regions of homology used for Gibson cloning of oligonucleotides are shown in purple. **c** Gene disruption using integrated pCas9-T2A-HXGPRT. RHΔ*hxgprt* and PruΔ*hxgprt* were transfected with vectors targeting *UPRT* or a control gene, *GRA29*. Transfectants were grown under M/X selection for 6 days before performing a plaque assay under 5-fluorodeoxyuridine (FUDR) selection to test for *UPRT* disruption. The percentage of *UPRT* knockout (KO) was calculated by comparing plaques numbers in the presence and absence of FUDR. The mean is shown with SD error bars. *n* = 2 biologically independent experiments. **d** Parasite survival is reduced after transfection of plasmid pools. RHΔ*hxgprt* and PruΔ*hxgprt* were transfected with 10 μg pCAS9-T2A-HXGPRT targeting *UPRT* or a mixed pool of 1000 gRNA. Parasites were grown in the presence of M/X and the resultant plaques counted after 7 days. The percentage of parasites surviving compared to parasites seeded was calculated in a plaque assay. The mean is shown with SD error bars. *n* = 3 biologically independent experiments. See also Supplementary Fig. 2. Source data are provided as a Source Data file

To expand the application of CRISPR screening in *Toxoplasma*, we designed a system where customised vector libraries can be generated by Gibson cloning pools of gRNA sequences into a common targeting vector. Single-stranded oligonucleotides encoding gRNAs are arrayed in multiwell plates, and can be individually selected to use directly for cloning without the need for hybridisation or amplification steps. The resulting vector library is transfected into parasites to generate a mutant pool that is propagated in cell culture for subsequent selection strategies in vitro or in vivo (Fig. 1a).

While the previously published *Toxoplasma* genome-wide guide library targeted the highly virulent type I strain^8^, type II strains are most commonly used for in vivo studies. We therefore designed gRNAs against the full *Toxoplasma* ME49 (type II) genome using E-CRISP^15^, and refined the list based on the criteria in Supplementary Fig. 1a, to generate 3–5 optimal gRNAs per gene (Supplementary Data 1). To account for the possibility of alternative start codons, gRNAs were designed to avoid the first 100 bp, and to reduce truncation (vs. complete KO), gRNAs were restricted to the first half of the gene. gRNAs targeting the non-template strand were favoured so the library could also be used for CRISPRi experiments^16^. gRNAs targeting 7616 genes were designed, 7183 genes with 5 gRNAs, and 211 and 222 genes with 4 gRNAs and 3 gRNAs, respectively (Supplementary Fig. 1b). ME49 gRNA sequences were aligned with the type I GT1 genome to identify those that can be used in both strains (Supplementary Data 1).

The previous *Toxoplasma* CRISPR screen used a parasite line stably expressing Cas9 and a mock guide to limit Cas9 toxicity, which was then transfected with the genome-wide gRNA library^8^. While this elegantly circumvents the problem of Cas9 toxicity, we reasoned that expressing the gRNA and Cas9 from a single vector would both eliminate the need for a mock gRNA, and expand the range of parasite strains in which this technique could be applied. We therefore generated a modified CRISPR vector by cloning a ribosomal skip peptide (T2A) and HXGPRT drug selection marker in-frame with Cas9-GFP (green fluorescent protein) (Fig. 1b). As expression of the selectable marker is linked to Cas9 expression, integration of the gRNA-expressing cassette without simultaneous integration of the Cas9 sequence is precluded. We introduced a modified tracrRNA sequence previously found to enhance the stability of the Cas9–gRNA interaction and improve gene targeting^17^. The resulting vector pCAS9-T2A-HXGPRT contains *Pac*I and *Nco*I restriction sites at the gRNA locus to allow single-step cloning of single-stranded gRNA-encoding oligonucleotides. Library generation is highly reproducible as shown by the correlation of sequencing reads from duplicate cloning reactions (*R*^2^ of 0.9994) (Supplementary Fig. 2A).

To validate the optimised CRISPR plasmid, we targeted the *UPRT* gene for disruption. Parasites lacking *UPRT* are able to survive in the presence of 5-fluorodeoxyuridine (FUDR). We tested KO efficiency in both a type I strain, RHΔ*hxgprt* (commonly used for cell culture experiments), and a type II strain, PruΔ*hxgprt* (used more frequently for in vivo experiments). Robust KO of *UPRT * was observed in both RHΔ*hxgprt* (89%) and PruΔ*hxgprt* (82%), while no parasites transfected with a control gRNA targeting *GRA29* grew in the presence of FUDR (Fig. 1c). While transfection efficiency was high (up to 40%), survival rates in transfected populations were low (1.3% in RHΔ*hxgprt* and 5.3% in PruΔ*hxgprt* (Fig. 1d)). Additionally, the transfection of pCAS9-T2A-HXGPRT plasmid pools showed a substantial reduction in parasite viability compared to a single plasmid transfection (RHΔ*hxgprt* 0.25%, PruΔ*hxgprt* 0.72% survival) (Fig. 1d). We hypothesised that this difference might be caused by the uptake of multiple plasmids in pooled transfections, resulting in multiple double-stranded DNA breaks that the parasites fail to repair. To investigate this, we performed co-transfections of plasmids targeting two different, non-essential genes (*GRA29* and *UPRT*) with either GFP or mCherry fused to Cas9, and quantified single and double fluorophore expressing parasites. At 24 h post-transfection of RHΔ*hxgprt*, 65% of parasites were expressing both fluorophores, which was reduced to 18% after 6 days of drug selection (Supplementary Fig. 2b). A similar pattern was observed with PruΔ*hxgprt* (57% at 24 h and 22% after 6 days) (Supplementary Fig. 2d). While many of these double transfectants will have died, 36% of RHΔ*hxgprt* and 25% of PruΔ*hxgprt* expressing mCherry only showed loss of GRA29, which was targeted by the GFP-expressing plasmid (Supplementary Fig. 2C, D). This indicates that in pooled transfections, parasites take up multiple gRNA plasmids causing gene deletions. However, often only one plasmid is retained and multiple KO events in the same cell will therefore not be detected by sequencing. While this did not prevent the generation of reproducible results in our experiments (as shown further below), it will be a general feature of pooled transfection strategies. As this impacts parasite survival, it is important that the number of parasites required to ensure sufficient coverage (i.e. parasites per gRNA) is calculated using library-specific post-transfection survival rates, and not standard transfection efficiencies. Using the latter would lead to a gross underestimate of the parasite numbers needed.

### Reproducible gene disruption using pooled gRNA libraries

To assess the reproducibility and the impact of library size and composition on phenotype scoring, we generated libraries of 210, 810 and 3231 gRNAs (referred to as 200, 800 and 3200 for simplicity, see Supplementary Data 2). The 200 gRNA library targets 33 genes encoding non-essential proteins that are predicted to be secreted, along with 5 essential controls^8^. The 800 gRNA library, meanwhile, is composed of the 200 gRNA library supplemented with gRNAs targeting 110 putative or known Toxoplasma effector proteins. These effectors are rhoptry proteins (ROPs) and dense granule proteins (GRAs), and are secreted into the parasitophorous vacuole (PV) or into the host cell. The majority of ROPs and GRAs are non-essential when grown in fibroblasts^8^. To test the impact of skewing the library towards fitness-conferring genes, we expanded the 800 library to target 466 proteins with a signal peptide or a single transmembrane domain (as these contain an increased proportion of essential genes), generating the 3200 library. Using the Sidik et al.^8^ phenotype scores associated with these libraries confirms that the 3200 library has a more negative median score than the 200 and 800 libraries (Supplementary Fig. 3). Each gRNA library was cloned into the pCAS9-T2A-HXGPRT vector, transfected into PruΔ*hxgprt* (in triplicate) and grown for 6 days to select for successful integration of the vector. Relative log 2 fold changes (lfcs) are calculated by comparing gRNA sequence reads from DNA input and after 6 days of parasite growth in human foreskin fibroblasts (HFFs). The median of the gRNA lfcs are used to calculate the gene lfc. This ensures that outliers do not substantially skew results. The final gene phenotype score is calculated by taking the average of the gene lfc across replicates (Supplementary Data 2).

**Fig. 2:**
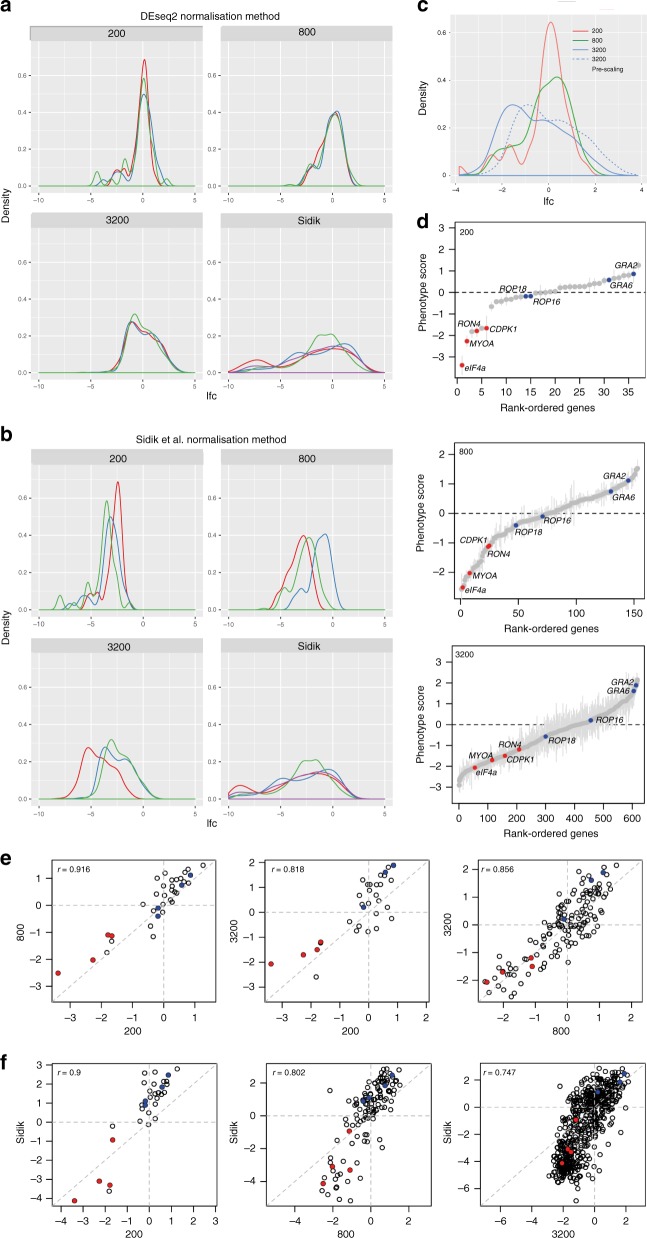
Variable pool sizes produce reproducible CRISPR selection results. PruΔ*hxgprt* were transfected with pCAS9-T2A-HXGPRT carrying 200, 800 or 3200 gRNA in triplicate transfections. The log 2 fold change (lfc) of the gRNA presence after 6 days growth in M/X compared to the DNA input is shown. Normalisation of sequencing counts using either **a** DESeq2 or **b** total read count for the 200, 800 and 3200 libraries and the published Sidik et al.^8^ dataset. Density plots show improved reproducibility between replicates for small libraries using DESeq2. **c** Overlay of 200, 800 and 3200 library gene lfcs with (solid line) and without (dotted line) centering of the 3200 library. **d** Ranked-order plots showing gene lfcs for 200, 800 and 3200 gRNA libraries. Essential and non-essential control genes are marked in red and blue, respectively. Error bars represent SEM. *n* = 3 technical replicates. **e**, **f** Correlation plots with Pearson’s correlation coefficient, *r*, of gene lfcs show that phenotypes are reproducible **e** across library sizes and **f** correlate well with published phenotype scores. Essential and non-essential control genes are marked in red and blue, respectively. See also Supplementary Fig. 3 and Source data in Supplementary Data 2

While Sidik et al.^8^ normalised the total read count per sample for their genome-wide CRISPR screen, we reasoned that with smaller library sizes, individual gRNAs that are highly over- or under-represented may strongly influence normalisation. We therefore used DEseq2^18^, which uses the median of ratios for normalisation (the median of each gene’s ratio to the geometric mean of the sample) and, as such, is less affected by outliers. Indeed, DEseq2 shows high correlation between the triplicate transfections for each pool (Fig. 2a), while the total read count approach does not (Fig. 2b). However, both methods give comparable results for the Sidik et al.^8^ genome-wide library (Fig. 2a, b). We therefore recommend using DEseq2 for normalisation of smaller libraries, but for larger libraries either method provides reproducible results. We then used the correlation between datasets to assess parameters that correlate with non-reproducible phenotype scores, such as sequencing read counts and the median absolute deviation (MAD) across gRNA. This analysis revealed that using the median of gRNA lfcs generated robust data, and that removing genes with the highest 1.5% of MAD scores would maximise correlations across datasets while retaining most genes. These results show that the use of dataset-appropriate normalisation and MAD thresholding provides robust fitness score calculations for small-scale libraries.

When comparing lfcs between the 200, 800 and 3200 libraries, we observed a shift in the 3200 compared to the 200 and 800 (Fig. 2c). This is likely a reflection of the DEseq2 normalisation, which assumes that the majority of a population do not change and centres on the median. Thus, the proportion of essential genes in a library can affect the positioning of zero lfc. While this has no bearing on the comparison of two conditions with similar median lfcs, it needs to be taken into account for experiments where a substantial proportion of gene lfcs change and shift the median. To allow for an accurate comparison between datasets we chose 25 genes present in our 3200 library that had a lfc close to zero (0 ± 0.2) in the Sidik et al.^8^ dataset as a reference point. We then shifted the lfc from the 3200 library such that the median score of these 25 gene lfcs was set to zero. This correctly aligned the lfcs across library sizes (Fig. 2c).

Ranked plots of gene lfc*s* demonstrated that control essential genes (*CDPK1*, *eIF4a*, *RON4* and *MYOA*) displayed the expected negative lfcs, while non-essential controls did not (Fig. 2d). The library size did not substantially influence gRNA behaviour (Fig. 2e) and our data correlated well with the Sidik et al.^8^ phenotypic scores (although differing lfc ranges between datasets skews the correlation axis away from the diagonal). This demonstrates that our optimised 3–5 gRNAs per gene are sufficient for reproducible CRISPR screening (Fig. 2f).

### In vivo CRISPR screening identifies novel virulence factors

**Fig. 3:**
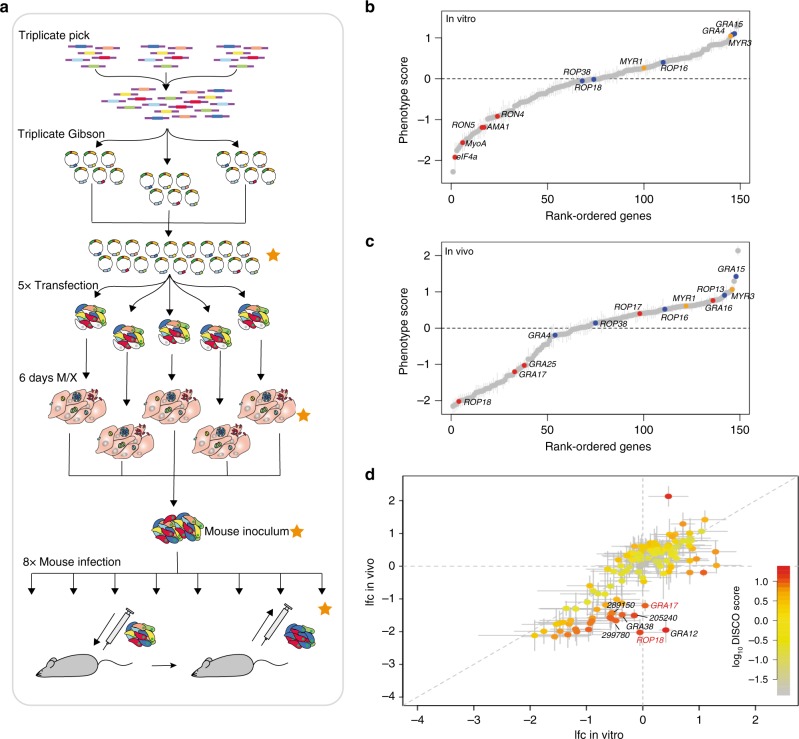
In vivo CRISPR screen identifies known and novel virulence factors. **a** Schematic of experimental design. gRNA were picked in triplicate, combined and used in triplicate Gibson cloning reactions. Vector pools were combined and transfected into PruΔ*hxgprt* in five replicates. After 6 days under M/X selection, subsets were taken for sequencing and remaining parasites were combined to generate the mouse inoculum. Eight mice were infected intraperitoneally (i.p.) with 200,000 parasites and the parasites retrieved from the peritoneum after 5 days. Orange stars indicate samples analysed by sequencing. **b**, **c** Rank-ordered plot of gene lfcs in vitro (**b**) and in vivo (**c**). Essential and non-essential control genes for in vitro and in vivo growth marked in red and blue, respectively. Translocon components MYR1 and MYR3 marked in orange. Error bars represent SEM. *n* = 5 technical replicates. **d** Discordance plot showing gene lfcs in vitro and in vivo. Colour indicates strength of discordance score. Error bars show 95% confidence range. Examples of known and newly identified virulence factors labelled with red and black text, respectively. See also Supplementary Fig. 4 and Source data in Supplementary Data 3

In pathogens, virulence factors that facilitate the interaction with the host determine whether or not a successful infection is established. To test whether we can infect mice with customised pools of *Toxoplasma* mutants, we integrated a ~1000 gRNA library into the type II strain PruΔ*hxgprt*. The resulting mutant pool was subsequently inoculated into mice at a dose of 2E5 parasites per mouse. We reasoned that this dose would allow parasite growth without overwhelming the mouse, while maintaining sufficient coverage per gRNA (>200 parasites/gRNA). The library included gRNAs targeting 16 essential genes, in addition to 133 known (ROPs and GRAs) and predicted effector proteins^19^. The gRNA library was cloned and transfected into parasites in five replicates (Fig. 3a). Following 6 days of selection in cell culture, genomic DNA from a subset of each of the replicates was isolated for sequencing, and the remaining parasites were pooled for intraperitoneal (i.p.) infection of eight mice. Five days post infection, the peritoneal exudate was retrieved and returned to tissue culture for parasite expansion, genomic DNA extraction and sequencing.

High reproducibility was observed between the five independent transfection replicates in vitro, as well as the eight independent infections in mice (Supplementary Fig. 4A and Supplementary Data 3). As expected, the 118 genes also contained in the 800 gRNA library showed high correlation of in vitro lfcs with the previous experiments (Supplementary Fig. 4B). Ranked-order plots showed that controls behaved as expected in both in vitro and in vivo experiments (Fig. 3b, c). The non-essential controls *GRA4*, *ROP38*, and *GRA15* showed expected phenotypes, with over-representation of gRNA targeting *GRA15* consistent with enhanced growth in vivo^20^. *ROP16* showed no difference in this type II strain in either cell culture or in vivo, while *ROP18*, *GRA25* and *GRA17* showed a negative fitness score in mice. However, contrary to previous reports, *GRA16* showed no reduction in vivo^21^. Moreover, MYR1 and MYR3, which form a translocon required to transport effectors (including GRA16) into the host cell^22,23^, similarly showed no phenotype in vivo. In addition, ROP17, which functions in translocation of effector proteins via the MYR complex^24^, showed no fitness cost in our in vivo screen. Collectively, this screening provides strong genetic evidence that translocation of MYR-dependent proteins into the host cell is not essential for the survival of individual parasites within the mouse peritoneum when represented in a pool.

To identify novel genes that are important for *Toxoplasma* proliferation in vivo but not in cell culture, we compared the gene fitness scores between in vitro and in vivo samples by calculating matched gene concordance/discordance (DISCO) scores (Fig. 3f and Supplementary Data 3). This analysis revealed previously unreported roles for secreted proteins in vivo when compared to their in vitro phenotypes. gRNAs targeting the dense granule proteins *GRA12* and *GRA38* were under-represented after 5 days of growth in the mouse peritoneum compared to the inoculum, while not required for in vitro parasite growth. Similarly, the previously unidentified *TGME49_205240*, *TGME49_299780* and *TGME49_289150*, were identified as contributing factors to in vivo fitness. In addition, although *GRA4* has a modest negative lfc in vivo (−0.194), the in vitro (1.078) and in vivo scores are discordant, suggesting that it does indeed contribute to in vivo parasite growth.

### Different requirements for *MYR1* in mutant pools vs. single KO

**Fig. 4:**
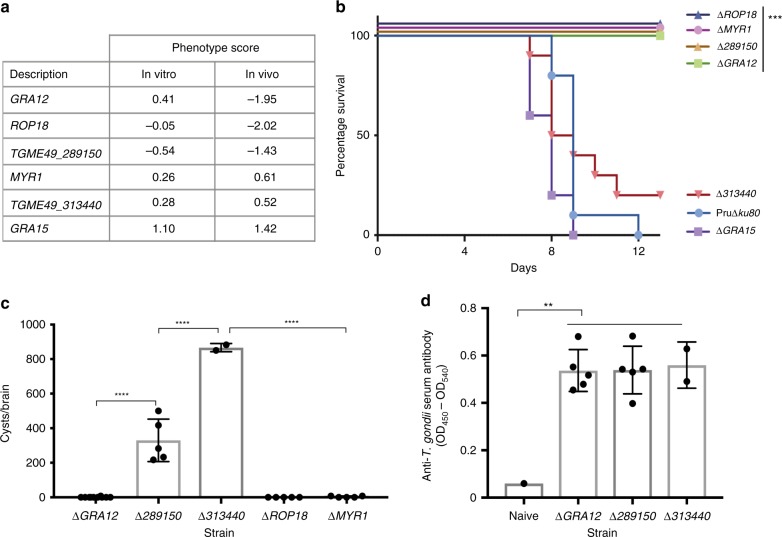
Confirmation of virulence defects identified in the CRISPR pool in vivo. **a** In vitro and in vivo phenotype scores for genes targeted for knockout (KO) and mouse infection. **b** C57BL/6 mice were infected intraperitoneally (i.p.) with 50,000 tachyzoites of the parental line PruΔ*ku80* (two experiments; *n* = 10 mice) and Δ*GRA12* (two experiments; *n* = 10 mice), Δ*TGME49_289150* (one experiment; *n* = 5 mice), Δ*TGME49_313400* (two experiments; *n* = 10 mice) and control strains Δ*GRA15* (one experiment; *n* = 5 mice), Δ*ROP18* (one experiment; *n* = 5 mice) and Δ*MYR1* (one experiment; *n* = 5 mice). Δ*ROP18*, Δ*MYR1*, Δ*TGME49_289150* and Δ*GRA12* were significantly different from PruΔ*ku80* with *P* values of 0.0075, <0.0001, 0.0004 and <0.0001, respectively, using the Mantel–Cox test. **c** Brains were isolated from surviving mice and cysts enumerated at 24 or 28 days post infection. Δ*TGME49_313400* *n* = 2, Δ*GRA12 n* = 10, Δ*TGME49_289150 n* = 5. *P* values were <0.0001 as calculated by one-way analysis of variance (ANOVA) with Tukey’s multiple comparison test. **d** Serum was isolated from mice surviving acute infection and tested for antibodies against *Toxoplasma-*soluble antigens in an ELISA. *P* values were Δ*GRA12* *P* = 0.006, Δ*TGME49_289150* *P* = 0.0058 and Δ*TGME49_313400* *P* = 0.0009 compared to the naive sample as calculated by one-way ANOVA with Tukey’s multiple comparison test. ** refers to *P* < 0.01, *** to *P* < 0.001 and **** to *P* < 0.0001. See also Supplementary Figs. 5 and 6. Source data are provided as a Source Data file

To validate the novel virulence factors identified and investigate the lack of an in vivo phenotype for MYR1 in our pooled infection, we introduced gene KOs in PruΔ*ku80* (Supplementary Fig. 5). We generated KOs of *GRA12* and *TGME49_289150*, predicted to be essential in in vivo infection from the screen, and *TGME49_313440* and *GRA15* as non-essential controls (summarised in Fig. 4a). We also generated KOs of *MYR1* and *ROP18* in PruΔ*ku80* to compare the pooled screen phenotypes with that of a homogenous gene KO. C57BL/6 mice were infected with 50,000 parasites and their survival monitored over 14 days. While mice infected with the parental line or the controls Δ*GRA15* and Δ*TGME49_313440* lost weight and succumbed to infection (10/10, 5/5 and 8/10, respectively), all mice survived Δ*GRA12*, Δ*TGME49_289150* and control Δ*ROP18* infections (Fig. 4b, Supplementary Fig. 6). Additionally, the Δ*MYR1* line was avirulent as previously reported^23^, confirming the contrasting phenotypes in pooled and single strain infections (Fig. 4a). Mice that survive acute *Toxoplasma* infection develop chronic infection with parasite cysts found in the brain. Analysis of the brains of surviving mice from this experiment revealed a lack of cysts in mice infected with Δ*GRA12*, and a reduced number of cysts in those infected with Δ*TGME49_289150* compared to the two mice surviving Δ*TGME49_313440* infection (Fig. 4c). As the inocula were shown to be comparable (Supplementary Fig. 6), and as enzyme-linked immunosorbent assay (ELISA) confirms all mice were infected (Fig. 4d), this validates GRA12 and TGME49_289150 as major virulence factors in mice, although the phenotype of GRA12 appears to be more pronounced.

Collectively, these results show a paramount role for ROP18, GRA12, and TGME49_289150 in *Toxoplasma* survival in both heterologous and homogenous KO populations. In contrast, MYR1, and by extension MYR1-dependent factors (MYR complex components and translocated proteins), appear only important in homogenous KO populations. This suggests a profoundly different role for these different effector proteins in host–pathogen interaction.

## Discussion

Here we present a method to perform tailored in vitro and in vivo CRISPR screens and demonstrate its potential by identifying novel virulence factors in *Toxoplasma*. We show that tailored CRISPR gRNA vector pools of various sizes can be readily and reproducibly generated by cloning single-stranded gRNAs into a vector carrying Cas9 and a selectable marker. We demonstrate that this strategy enables us to target different parasites strains using a range of different library sizes. As such, gRNA pools targeting only a subset of genes, such as transcription factors or the kinome, can be easily generated to address specific biological questions.

While we have established the system using the *HXGPRT* selection marker, which requires a Δ*hxgprt* background strain, using an alternative marker (e.g. *DHFR*) would further broaden the use of the system. Importantly, this would mean that genetic screening could be applied to exotic *Toxoplasma* isolates to address the specific host–pathogen interactions that permit disease in immunocompetent hosts.

We have uncovered limitations in this system that are likely true for other pooled transfection-based screens, caused by the occurrence and resulting lethality of multiple CRISPR plasmids entering parasites simultaneously. This is important information when designing screens, as it informs coverage calculations for experimental setup. It will also need to be taken into consideration when single-cell analysis experiments with mutant pools are analysed or under strong selective pressure. For example, survival screens under drug pressure, which could select for mutants for which the gRNA plasmid is not retained. Additionally, some care must be taken with phenotypes from large datasets. For example, the secreted protein GRA36 is flagged as having an in vivo phenotype, but careful analysis of the gRNA revealed that discrepancies between genome annotation versions mean these gRNA actually target a neighbouring non-secreted protein. Despite these limitations, we have reproducibly identified known essential controls in vitro and in vivo and identified novel virulence factors.

Surprisingly, this screen separated known effector proteins into two groups: those genes required for in vivo growth within a population of mutants (such as *ROP18*, *GRA17* and *GRA25*) and those that are required for growth in vivo when infecting as a single mutant, but not when part of a mutant pool (such as *MYR1*, *MYR3*, *ROP17* and *GRA16*). Intriguingly, many of the factors identified here as required for in vivo infection localise within the PV or at the vacuole membrane (ROP18, GRA12, GRA17, GRA25, GRA38, GRA39). Those factors not required for survival in the peritoneum within a pool are either required for translocation of parasite proteins into the host cell or are translocated effectors. ROP18 has multiple functions within an infected cell (targeting immunity regulated GTPases (IRGs), ATFb6, and NFΚB) and is known to promote parasite survival^25–28^. Crucially, it directly phosphorylates host IRGs preventing destruction of the vacuole within murine cells, although this phenotype is more pronounced in type I strains^26,27^. GRA17, meanwhile, is required for parasite fitness by forming a pore in the vacuole membrane for nutrient access^29^, and GRA25 deletion causes differential cytokine secretion of infected macrophages^30^. GRA12 localises within the PV^31^, and its strong phenotype in the screen resembling that of ROP18 suggests a role in parasite survival or cell-autonomous immunity. Reassuringly, while this manuscript was under review, Fox et al.^32^ identified an important function for GRA12 at the PV in the resistance of *Toxoplasma* to IRG mediated death^32^. Furthermore, the recently identified GRA38 and GRA39 localise within the PV^33^ and show virulence defects in our study, whereas the MYR1-translocated effector TEEGR/HCE1 does not^34,35^.

ROP17, which appears to play a role in a ROP18/ROP5 complex acting on IRGs in the highly virulent type I strain^36^, does not contribute to IRG defences in a type II strain and shows a dose-dependent requirement for virulence^37^. Recently, ROP17 has been shown to be required for translocation of proteins via the MYR complex^24^ and consistently does not appear to be important in vivo in our study when part of a heterogenous CRISPR mutant population in type II parasites. This finding further supplements the power of our CRISPR strategy as it can be used in all three major *Toxoplasma* strains to uncover strain-specific differences in the future.

How the newly identified TGME49_205240, TGME49_289150 and TGME49_299780 function in *Toxoplasma *biology requires further clarification. As we have not further investigated TGME49_205240 and TGME49_299780 in this study, their role in virulence needs to be confirmed. TGME49_205240 is a multipass-transmembrane domain protein with a predicted Cleft Lip and Palate domain of unknown function. TGME49_289150 and TGME49_299780 have no predicted functional domains. TGME49_289150 is present with high sequence identity (>98%) and shares synteny across the canonical type I, II and III *Toxoplasma* reference strains. Although no signal peptide is predicted for TGME49_289150, an internal single transmembrane domain could be functioning as a recessed signal as is the case for GRA6, GRA37 and GRA40^33,38^. TGME49_289150 was also shown to be phosphorylated only when inside host cells, a hallmark of many proteins secreted into the host cell^19^. Its modest phenotype compared to GRA12 and ROP18, however, suggests that it is not absolutely required for the IRG defence pathway. Regardless of its mechanism, deletion of TGME49_289150 impairs the ability of *Toxoplasma* to mount a lethal infection at the doses used, supporting the in vivo CRISPR screening data.

The observation that GRA16 (which targets p53 signalling^21^ and the MYR translocation complex, required for the majority of transcriptional changes in the host cell^39^) were not required in a pooled infection warrants further investigation. One hypothesis is that the MYR1-dependent effectors become important later during the disease progression, for example, during dissemination. However, unlike wild-type (WT) parasites, *MYR1* KOs do not lose weight during the onset of infection, indicating that these mutants never establish an infection, which argues against this hypothesis. More likely is the hypothesis that MYR1-dependent effectors control the overall immune environment within the host. In this case, parasites within the pool that do not carry deletions for MYR1-dependent effectors could still exert this function, allowing MYR1-dependent effector mutants to piggyback. The substantial transcriptional changes in the host cell that are caused by MYR1-dependent effector proteins^39^, some of which are counterbalancing, make it hard to predict what immune signalling may contribute to the trans-rescue of MYR1 mutants in the pool. Targeted studies to understand why the different effector proteins are separated in the in vivo CRISPR screen will likely illuminate the survival strategies of *Toxoplasma* in its murine host. Future studies aim to investigate the role of MYR1 and its dependent proteins compared to those known to act in the IRG response (ROP18, ROP5, GRA12) in *Toxoplasma* survival. Whatever the distinction between these effector classes, our results suggest that their biology can be teased apart in this kind of pooled screening. It also suggests that this strategy may complicate the identification of MYR1-dependent effectors in in vivo screens. However, as MYR1 has been shown to have profound transcriptional effects on the host cell, one could use single-cell transcriptomics of a host cell infected with mutant pools, which we are currently establishing.

Within the field of *Toxoplasma* infection, our CRISPR screening method will allow the identification of genes required for survival of different parasite strains in a variety of hosts, as well as epistasis studies to identify genetic interactions between genes, to name but a few. Furthermore, with an expanded library of putative effectors, we predict to be able to identify more virulence factors in the future. While we have established this method in *Toxoplasma*, the format of arrayed single-stranded oligonucleotides for versatile pooled gRNA libraries and the analysis of smaller library screens will be advantageous in a range of experimental setups and model systems.

## Methods

### Cell culture and parasite strains

Primary HFFs (ATCC) were maintained in Dulbecco’s modified Eagle’s medium (DMEM) with 4.5 g/L glucose and GlutaMAX-1 (Gibco) supplemented with 10% foetal bovine serum at 37 °C and 5% CO_2_. *Toxoplasma gondii* strains RHΔ*hxgprt*^40^, PruΔ*hxgprt* (gift from Dominique Soldati, as in ref. ^40^) and PruΔ*ku80* ^41^ were maintained by growth in confluent HFFs and passaged every 2–3 days. Parasites were seeded 2 days prior to transfection for PruΔ*hxgprt* and *Pru*Δ*ku80*, or 1 day for RHΔ*hxgprt*.

### Plasmid construction

pCAS9-T2A-HXGPRT was generated by introducing *Thosea asigna* virus 2A-like sequence (T2A)^42^ and *Toxoplasma HXGPRT* sequences into pSAG1::CAS9-U6::gUPRT^43^. All primer sequences are shown in Supplementary Table 1. *HXGPRT* was amplified from pGRA^44^ with primers 1 and 2, and combined at equal ratios with 50 ng *Pac*I-digested pSAG1::CAS9-U6::gUPRT, and an oligonucleotide containing the T2A sequence (primer 3) by Gibson assembly in a homemade reaction master mix (100 mM Tris-Cl, pH 7.5, 50 mg/ml PEG-8000, 10 mM MgCl_2_, 10 mM dithiothreitol, 0.2 mM each of four dNTPs, 1 mM NAD with 0.8 U T5 exonuclease (NEB), 0.5 U *Phu* DN polymerase (NEB) and 80 U *Taq* DNA ligase (NEB)). The Gibson reaction was incubated for 60 min at 50 °C. Inverse PCR was used to insert *Pac*I and *Nco*I restriction sites into the gRNA expression locus and modify the tracrRNA sequence using primers 3 and 4 and 5 and 6, respectively. gRNA sequences were introduced into pCAS9-HXGPRT and pSAG1::CAS9 vectors by inverse PCR with primer 4 and gRNA-specific primers 5 and 32–37. To generate an mCherry-tagged Cas9 vector, pCAS9-T2A-HXGPRT::gUPRT excluding the GFP sequence was amplified (primers 10 and 11) and combined with an mCherry sequence with 20 bp of homology in a Gibson reaction as above. Plasmid sequences were verified by sequencing.

### gRNA library generation

Twenty base pair gRNA sequences were designed against the *Toxoplasma* ME49 genome (v7.1.31) using E-CRISP^15^ command line version cld-1.4.0 2017-01-19. gRNA were filtered as per the criteria in Supplementary Fig. 1 to select 5 per gene. The criteria were dropped sequentially and the positioning parameter relaxed from 50 to 60% of coding sequence where needed to generate 3–5 gRNA per gene. For comparison with the *Toxoplasma*-type I GT1 genome, the ME49 guides for each gene were extracted from the generated large.tab file and then compared to the ToxoDB release 41 of the GT1 genome using the program fuzznuc from the EMBOSS suite (6.6.0) with mismatches from 0 to 3. All of these fuzznuc files were collated into a table with the use of a custom perl script to parse the data.

Single-stranded DNA oligonucleotides including gRNA sequences with 20 bases of homology to either side of the pCAS9-T2A-HXGRPT integration site (Supplementary Table 1) were ordered from Sigma-Aldrich. Note that the reverse complement was ordered to avoid a quadruple G in the homology arm. Oligonucleotides were resuspended in 10 mM Tris-HCl (pH 8.0), 0.1 mM EDTA (TE) buffer to 100 μM and diluted to 0.1 μM in 384-well Echo qualified microplates (Labcyte). For gRNA pools, 7.5–20 nl (depending on pool size) oligonucleotides were picked in triplicate using the Echo 550 liquid handler (Labcyte). Combined gRNA picks were incubated with 100 ng *Nco*I/*Pac*I-digested pCAS9-T2A-HXGPRT at a ratio of 10:1 in Gibson reaction mix for 1 h at 50 °C. DNA was ethanol precipitated and resuspended in 3 μl H_2_O. A measure of 1.5 μl of DNA was transformed into 20 μl MegaX DH10B T1 R electrocompetent *E. coli* (Invitrogen) and allowed to recover for 1 h at 37 °C before overnight growth in 350 ml LB Ampicillin (100 μg/ml). Plasmid DNA was purified using Macherey-Nagel Midiprep kit and reconstituted in TE buffer.

### Parasite transfection

Input DNA (single plasmid or library pool) was digested overnight with *Kpn*I-HF (NEB), purified by phenol–chloroform precipitation and resuspended in P3 primary cell electroporation buffer (Lonza) to a concentration of ~1.5 μg/μl. Parasites were isolated from HFFs by syringe lysis with a 23 G needle, pelleted at 652 × *g*, and resuspended in P3 buffer. 7.5E5-2E6 parasites were combined with 10 μg linearised plasmid DNA in a total volume of 20 μl and electroporated using the 4D-Nucleofector EO115 program (Lonza). Parasites were allowed to recover for 12 min before being added to HFF monolayers (T150). At 24 h post transfection, 50 μg/ml of mycophenolic acid (Merck) and xanthine (Sigma) (M/X) were added to select for transfectants.

### Plaque assays

To test the proportion of parasites surviving transfection, 5000 parasites were seeded onto HFF monolayers in a T25 flask 12 min after transfection. After 24 h, M/X was added and plaques were allowed to form over 8 days. To test the proportion of *UPRT* KO, parasites were selected in M/X for 6 days post-transfection and 1000 parasites seeded in T25s in M/X alone or M/X with 20 μg/ml FUDR and grown for 6 days. For testing mouse inoculum, 10 μl of a 1:10 dilution of the inoculum was used to infect HFF monolayers in 6-well plates. Monolayers were fixed in ice-cold methanol for 2 min, stained with crystal violet (12.5 g crystal violet in 125 ml ethanol mixed with 500 ml of 1% ammonium oxalate) for 10 min and the plaques enumerated.

### Antibody generation

To generate the GRA29 expression plasmid, pET-28a-GRA29, *GRA29* lacking its amino-terminal signal peptide was amplified from *Toxoplasma* gDNA (prepared from 1E7 RHΔ*hxgprt* using Qiagen Blood and Tissue DNA extraction kit) using primers 30 and 31 (Supplementary Table 1). The amplicon was cloned into *Bam*HI- and *Nde*I-digested pET-28a(+) plasmid (Merck) by Gibson as described above. This allowed for expression of an N-terminal 6×His-tagged GRA29 recombinant protein in *E. coli* BL21 cells under the control of T7 *lac* promoter. His-GRA29 was purified using Ni-NTA affinity purification under native conditions using the standard manufacturer’s protocol (Qiagen, Hilden, Germany). Recombinant His-GRA29 was used to immunise female New Zealand white rabbits (Covalab) for the generation of polyclonal antibodies. To verify antibody specificity,* Toxoplasma* lysate was separated by sodium dodecyl sulfate-polyacrylamide gel electrophoresis, transferred onto nitrocellulose (Bio-Rad) and probed with serum at a dilution of 1:1000 in phosphate-buffered saline (PBS) with 3% milk and 0.1% Tween-20 (Sigma-Aldrich). After washing, the blot was incubated with horse radish peroxidase (HRP)-conjugated anti-rabbit secondary antibody (1:20,000, Insight Biotechnology, cat no. 474-1506). The blot was visualised using ECL Western Blotting Detection Reagent (GE Healthcare) for chemiluminescence imaging on an Amersham Imager 600 (GE Healthcare) and the band of the expected size was detected (Supplementary Fig. 2).

### Immunofluorescence

HFF monolayers were infected at a multiplicity of infection of 1 for 24 h and fixed in 3% paraformaldehyde for 15 min. Cells were permeabilised in 0.1% Triton for 5 min and blocked with 2% bovine serum albumin (BSA) before staining with rabbit anti-GRA29 (1:4000), followed by anti-rabbit Alexa Fluor 647 (1:2000, Life Technologies, cat. no. A21244) and DAPI (4′,6-diamidino-2-phenylindole) (1:1000 of 5 mg/ml, Sigma-Aldrich). GRA29 staining and Cas9-GFP/Cas9-mCherry expression were counted on a Ti-E Nikon microscope.

### In vitro and in vivo mutant pool selections

Four days post transfection, parasites were syringe lysed and added to fresh HFF monolayers with 100 U/ml benzonase (Novagen) and incubated overnight to remove traces of input DNA. Six days post transfection, samples were taken for gDNA preparation or for mouse inoculum. For gDNA samples, parasites were syringe lysed and passed through 5 μm filters (Millipore), counted and at least 1E7 parasites pelleted at 3220 × *g*. For mouse inoculum, parasites were filtered as above, pelleted at 652 × *g* and resuspended in PBS at a concentration of 1E6 parasites/ml.

C57BL/6 mice were bred and housed under pathogen-free conditions in the biological research facility at the Francis Crick Institute in accordance with the Home Office UK Animals (Scientific Procedures) Act 1986. All work was approved by the UK Home Office (project license PDE274B7D), the Francis Crick Institute Ethical Review Panel and conforms to European Union directive 2010/63/EU. Six- to eight-week-old C57BL/6 males were injected i.p. with 200,000 parasites in 200 μl PBS. Five days post infection, parasites and peritoneal cells were isolated by peritoneal lavage with 5 ml of 0.5 mM EDTA/PBS. Cells and parasites were pelleted at 652 × *g*, washed with PBS and added to HFF monolayers growing in DMEM with Pen/Strep (Thermo Fisher). Pellets for gDNA were prepared as above, after 4 days parasite growth.

### gRNA amplification and sequencing

gDNA was isolated using DNeasy Blood and Tissue kit (Qiagen) and resuspended in 100 μl H_2_O. gRNA sequences were amplified by nested PCR using Kapa HiFi HotStart polymerase (Roche). PCR 1 amplified a 4 kb fragment using primers 8 and 9 with sufficient template for 500× coverage per gRNA with a maximum of 2E5 parasites/PCR reaction in 25 μl reaction volume (e.g. for the 800 gRNA library, gDNA corresponding to 4E5 parasites was used as a template, needing 2× PCR 1 reactions). Twelve and 18 cycles were used for plasmid DNA input, and gDNA was isolated from parasites, respectively. The PCR product was purified using 0.6× vol of Kapa Pure beads (Roche), and 8 μl was used as a template in each of 6× of 50 μl nested PCR 2 reactions, with primers (10 with 12–23) including Illumina adaptor and index sequences. Twenty and 25 cycles were used for plasmid DNA input, and gDNA was isolated from parasites, respectively. Amplicons were purified as above and mixed at equal ratios (or taking library size into consideration) and sequenced on HiSeq4000 with paired end 100 bp reads using the HiSeq Control Software version 3.4.0.

### Illumina sequencing analysis

Data were analysed using DEseq2 v1.20.0, R v 3.5.2 and ggplot2 v 3.1.1. Computer code used in this study is available from the corresponding author upon reasonable request. Following demultiplexing, gRNA sequences were trimmed and matched to target library to assess gRNA representation. Sequencing counts were normalised using DEseq2^18^ with default settings across gRNAs that corresponded to genes that had at least three non-zero gRNAs. Thresholding for the maximum MAD across gRNAs was set by selecting the value that gave the highest Pearson’s correlation between libraries (testing 0–20%, with 0.5% step size). A threshold removing genes with the highest 1.5% of MAD scores were applied to all datasets. Gene lfcs were calculated per sample from the median of gRNA lfcs and the mean of gene lfcs across replicates gives the gene phenotype. The 3200 library was centred around zero by applying a scaling factor from published gene phenotypes^8^ with a value between −0.2 to 0.2 and standard error <0.5. Gene phenotypes between in vitro and in vivo conditions were compared in a concordance/discordance plot with a confidence (DISCO) score (DISCO score = abs(avg(lfc_in vitro_) − avg(lfc_in vivo_)) × abs(log 10 − *P* value + log 10 *P* value).

### KO generation

A Pro-GRA1::mCherry::T2A::HXGPRT::Ter-GRA2 construct was amplified with primers containing 40 bp homology regions to the 5′- and 3′-untranslated regions of the target genes (primers 38–49). Fifteen micrograms of pSAG1::CAS9 carrying gRNA against the target gene and 10 µg of purified PCR product were co-transfected into 10^6^–10^7^ PruΔ*ku80* tachyzoites. Twenty four hours after transfection, M/X was added to select for integration of the mCherry construct. Correct mCherry integration and loss of the WT locus were verified by PCR using primers 50–63.

### In vivo infections

Mice were infected i.p. with 50,000 parasites in 200 µl PBS on day 0. Mice were monitored and weighed regularly for the duration of the experiments. To determine the number of cysts in the brain of infected animals, mice were euthanised at 24–28 days post infection. The brain was homogenised in 1 ml PBS and stained with Rhodamine-conjugated *Dolichos Biflorus* agglutinin (1: 2000; Vector Laboratories, cat. no. RL-1032) for 1 h at room temperature. Fluorescently labelled cysts were counted using a Ti-E Nikon microscope. For serum samples, jugular vein blood was drained into blood serum collection tubes (SAI, Infusion technologies) and allowed to clot at room temperature for 30 min, before the tubes were centrifuged at 1500 × *g* for 10 min. Serum was collected and stored at −20 °C until analysis. Data were analysed in GraphPad Prism v7. Survival curve analysis was used with the Mantel–Cox test for each KO line compared to PruΔ*ku80*. For cyst count analysis, a one-way analysis of variance (ANOVA) with Tukey’s multiple comparison test was used.

### *Toxoplasma* serum antibody ELISA

To prepare *Toxoplasma*-soluble antigens (TSAs), parasites were syringe lysed, washed once with PBS and adjusted to 1 × 10^8^ tachyzoites/ml with PBS containing protease inhibitors (c*O*mplete mini, Roche). Parasites were lysed by five freeze–thaw cycles (liquid nitrogen/37 °C), followed by ultrasound sonication on ice (five 60 Hz cycle for 1 min each). Samples were centrifuged at 10,000 × *g* for 30 min at 4 °C before supernatants were collected and protein content was determined using the BCA Protein Assay kit (Pierce) following the manufacturer’s instructions. To detect *Toxoplasma* antibodies in murine serum samples, 96-well plates (flat bottom, high-binding) were coated overnight with 2 µg/ml TSA at 4 °C. Plates were washed with PBS/0.05% Tween-20 (v/v) (PBS-T) before blocking with 1% BSA (w/v) in PBS. Bound antigens were incubated with murine sera diluted 1/10 in 1% BSA/PBS, washed three times with PBS-T and bound antibodies detected by incubation with anti-mouse immunoglobulins (HRP conjugate, Dako, cat. no. P0260) diluted 1:1000 in 1% BSA/PBS. All incubation steps were 2 h at room temperature. Finally, plates were washed three times with PBS-T and developed by adding TMB substrate solution (Thermo Fisher). The TMB reaction was stopped by adding 2 N sulphuric acid and the absorbance was measured (OD_450_ minus OD_540_) using the VersaMax^TM^ Microplate Reader with the SoftMax^®^ Pro Software. Data were analysed in GraphPad Prism 7 with a one-way ANOVA with Tukey’s multiple comparison test.

### Reporting summary

Further information on research design is available in the Nature Research Reporting Summary linked to this article.

## Supplementary information

Supplementary Information

Reporting Summary

Description of Additional Supplementary Files

Supplementary Data 1

Supplementary Data 2

Supplementary Data 3

Source Data

## Data Availability

The authors declare that the data supporting the findings of this study are available within the paper and its supplementary information files. The source data for Figs. 1c, d, 4b–d and Supplementary Figs. 2a, c, 6a, b are provided as a Source Data File. All source data corresponding to CRISPR phenotype scores are RAW sequencing read counts which are found in Supplementary Data 2 and 3.

## Code availability statement

Computer code used in this study is available from the corresponding author upon request.
