# Supplementary material for: A CRISPR platform for targeted in vivo screens identifies Toxoplasma gondii virulence factors in mice
Source: Nat Commun. 2019 Sep 3;10:3963. doi: 10.1038/s41467-019-11855-w (PMC6722137; doi:10.1038/s41467-019-11855-w)
Supplement: Supplementary file 1 — Supplementary Information [file 41467_2019_11855_MOESM1_ESM.pdf]

Supplementary Information

A CRISPR platform for targeted *in vivo* screens identifies *Toxoplasma gondii* virulence factors in mice

Young *et al*

Supplementary Figure 1

a.

gRNA were designed using ECRISP and 3-5 selected per gene based on the following criteria

1. Minimal off target effects (including plasmid sequence) (already in ECRISP)
2. Minimal overlap between guides (already in ECRISP)
3. Poly T (already in ECRISP)
4. No KpnI site in sequence (as this is used to linearize plasmid prior to transfection)
5. Location:
   a. Avoid first 100 bp of gene
   b. No later than 50% of cds
   c. Not in UTRs
6. Intermediate GC content (between 40 and 80%).
7. Either G at -4 (from PAM) ; G/A at -2 (from PAM) ; G/A at -1 (from PAM)
8. Target non-template strand (for usage in CRISPRi screens)

b.

	**Number of genes with**		
**Number of criteria met**	**5 gRNA**	**4 gRNA**	**3 gRNA**
**8**	5879		
**7**	625		
**6**	53		
**5**	107		
**5 - 4 bp overlap**	130		
**5 - 60% gene, 4 bp overlap**	389	211	222

Supplementary Figure 1. Design of gRNAs. (a) List of criteria used for gRNA design and selection. Where this did not give >3 gRNA, criteria were dropped (from 8 downwards) and the stringency was relaxed allowing up the the first 60% of the coding sequence and 4 bp overlap between gRNAs. **(b)** Number of genes with gRNA designed showing the number of gRNA/gene and the number of criteria met.

Supplementary Figure 2

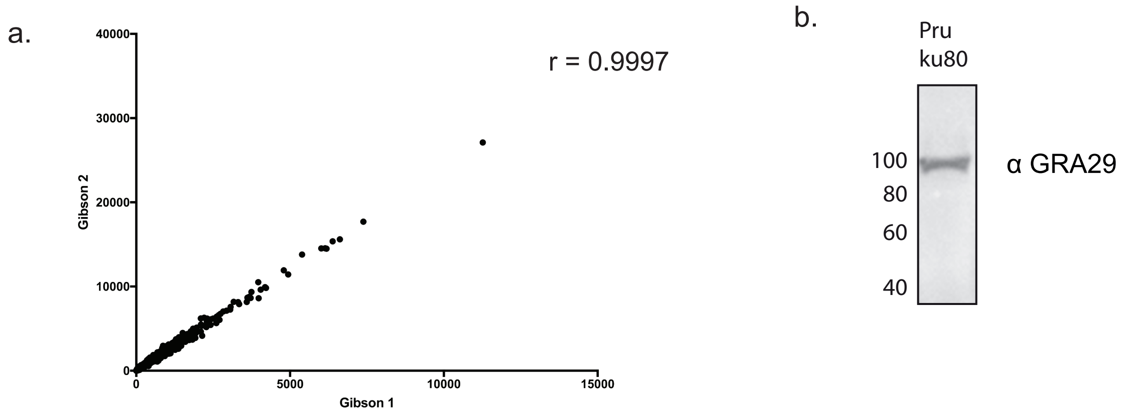

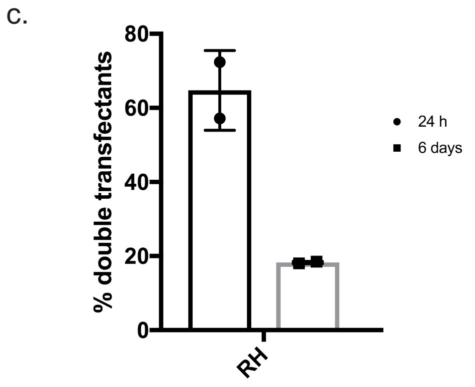

d.

	Timepoint	RHΔ*hpt* %	PruΔ*hpt* %
GFP+ MCh+	24 h	65 (2)	57 (1)
GFP+ MCh+	6 days	18 (2)	22 (1)
GFP- MCh + Gra29-	6 days	36 (1)	25 (1)

Supplementary Figure 2. Testing of cloning reproducibility of gRNAs and double transfection frequency (a) Gibson cloning of vector pools is highly reproducible. Correlation plot with Pearson correlation coefficient *r*, of sequencing reads from two independent cloning reactions of the 800 gRNA library. **(b,c)** Parasites take up multiple plasmids in mixed transfections. RHΔ*hxgprt* and PruΔ*hxgprt* were co-transfected with pCAS9(GFP)-T2A-HXGPRT targeting *GRA29* and pCAS9(mCherry)-T2A-HXGPRT targeting *UPRT* and the proportion of parasites expressing both fluorophores at 24h and after 6 days M/X selection were counted. **(b)** The mean is shown with SD error bars shown. n = 2 biologically independent experiments. **(c)** Immmunoblot showing a band of the expected size when *T. gondii* lysate was probed with the α-GRA29 antibody. **(d)** Table showing the percentage of parasites expressing both fluorophores and the percentage expressing mCherry only that were lacking staining for GRA29. Number of experiments shown in brackets.

Supplementary Figure 3

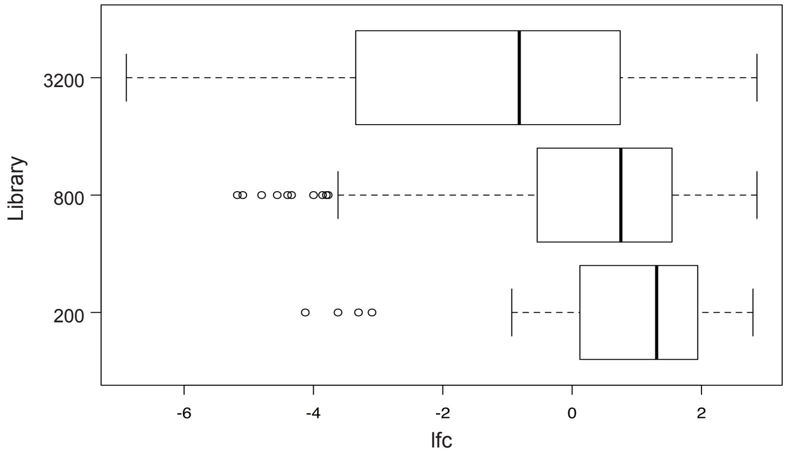

Supplementary Figure 3. The 3200 gRNA library targets a higher proportion of essential genes. Boxplots showing the median phenotype scores assigned by Sidik *et al* for the genes targeted in the 200, 800 and 3200 gRNA libraries [1]. Median and first quartiles are shown by the box, and 1.5x interquartile range indicated by whiskers. Outliers are shown as circles.

Supplementary Figure 4

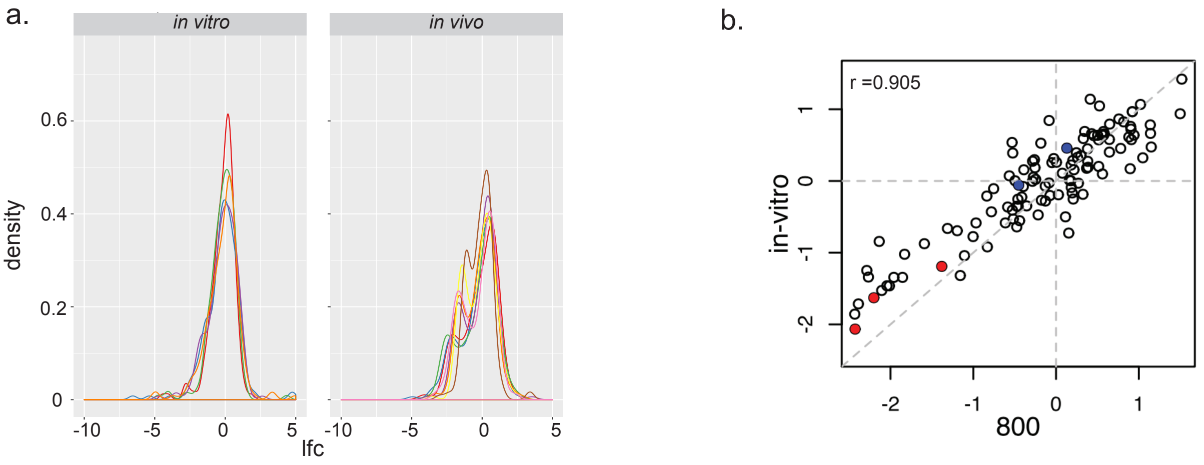

Supplementary Figure 4. Reproducible phenotype score distribution *in vitro* and *in vivo.*

(a) Overlay of lfcs for each replicate following DESeq2 normalisation for *in vitro* (n=5 technical replicates) and *in vivo* (n=8 mice) analysis. **(b)** Correlation plot with Pearson's correlation coefficient *r,* of gene lfcs compared to the 800 gRNA library showing high reproducibility between experiments. Source data in Supplementary Data files 2 & 3.

Supplementary Figure 5

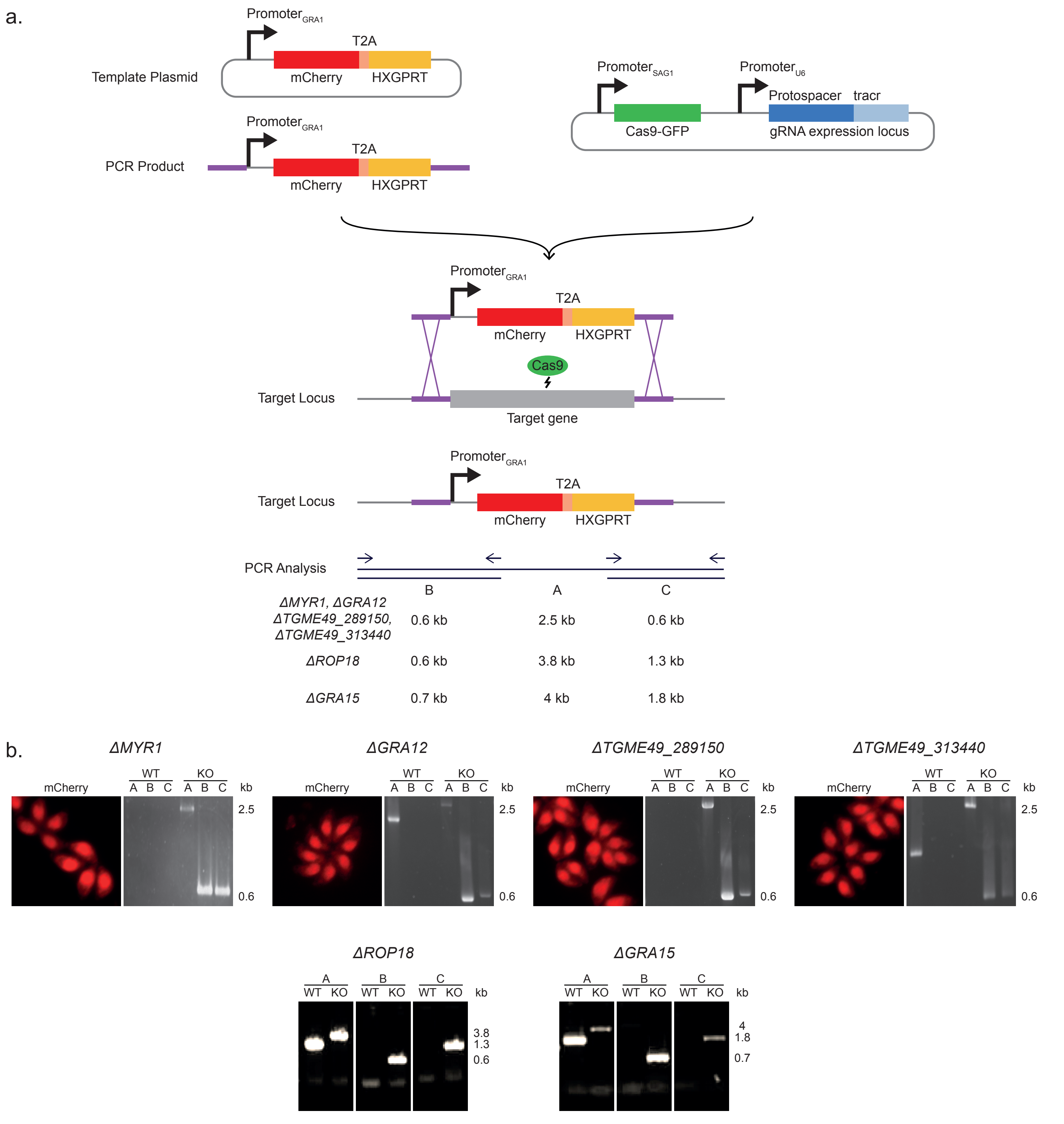

Supplementary Figure 5. Generation of gene knockouts (a) Schematic of the gene knockout strategy. mCherry flanked by GRA1 promoter and GRA2 3' UTR was amplified with primers adding homology to gene of interest. A double strand break was introduced by Cas9 to promote integration of mCherry and disruption of the locus **(b)** PCRs verifying correct integration of mCherry into *MYR1, GRA12,* TGME49_289150, TGME49_313440, *ROP18* and *GRA15* loci. A, B, C refer to amplicons indicated in **(a).**

Supplementary Figure 6

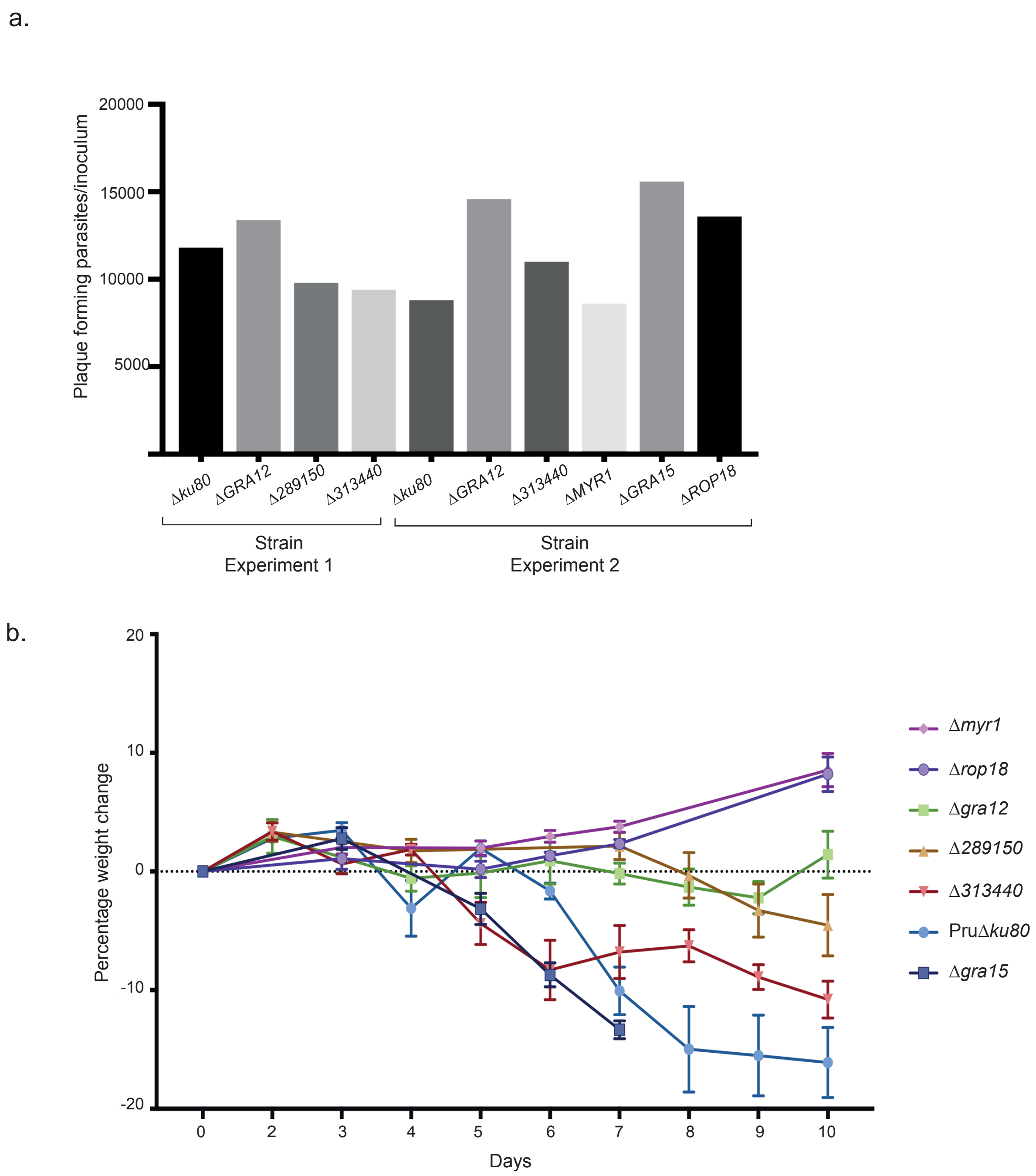

Supplementary Figure 6. *In vivo* phenotypes of single knock out mutants. Mouse inoculum and weight loss during infection. C57BL/6 mice were infected i.p. with 50,000 tachyzoites of the parental line Pru*ku80* (two experiments; n = 10 mice) and *ΔGRA12* (two experiments; n = 10 mice)*, ΔTGME49_289150* (one experiments; n = 5 mice), *ΔTGME49_313400* (two experiments; n = 10 mice), and control strains *ΔGRA15* (one experiments; n = 5 mice)*, ΔROP18* (one experiments; n = 5 mice) and *ΔMYR1* (one experiments; n = 5 mice). **(a)** Plaque forming parasites per mouse inoculum as calculated from the plaques formed from 10ul of a 1:10 dilution of the inoculum. **(b)** Mouse weight was monitored over the course of infection. Mean weight loss shown per group with error bars denoting SD.

Supplementary Reference

1. Sidik, S. M. *et al.* A Genome-wide CRISPR Screen in Toxoplasma Identifies Essential Apicomplexan Genes. *Cell* **166**, 1423-1435.e12 (2016).

Supplementary Table 1. Oligonucleotides used in this study. For the Illumina sequencing primers the barcode is shown in blue and spacers in red.

Number	Name	Oligonucleotide sequence
1	hxgprt (2A hr) F	catgtggagatgtagaagaaaatccaggaccacccgggatggcgtccaaacccattgaag
2	hxgprt (pCas9) R	tttgtcgatttgagaagtgagcacaacggtgagaattcttacttctcgaactttttgcg
3	PacI-NcoI gRNA F	ttaattaacgactcgaccatgggttttagagctagaaatagc
4	pSAG1::cas9UG R	aacttgacatccccatttac
5	gRNA GRA29 F	ttgaaacgagcaatgaaccagttttagagctagaaatagc
6	TracrRNA opt F_univ	cagcatagcaagtttaaataaggctagtcc
7	TracrRNA opt NcoI PacI R	tttccagcatagctcttaaacccatggtcgagtcg
8	TracrRNA opt UPRT R	tttccagcatagctcttaaacgctctcacaatcgagacg
9	TracrRNA opt GRA29 R	tttccagcatagctcttaaactggttcattgctcg
10	pCAS9-T2A-HXGPRT CAS9 F	tgatcatcaagctgcctaagtactc
11	pCAS9-T2A-HXGPRT 1481R	taatcctgttaccagtggctgc
12	MiSeq F1	aatgatacggcgaccaccgagatctacactctttccctacacgacgctcttccgatcttaagtagagtcacctcgtccccgaagg
13	MiSeq F1 suggested	aatgatacggcgaccaccgagatctacactgaaccttacactctttccctacacgacgctcttccgatctttcacctcgtccccgaagg
14	MiSeq R1	caagcagaagacggcatacgagataagtagaggtgactggagttcagacgtgtgctcttccgatcttagtcagtgagcgaggaagc
15	MiSeq R2	caagcagaagacggcatacgagatacacgatcgtgactggagttcagacgtgtgctcttccgatctatagtcagtgagcgaggaagc
16	MiSeq R3	caagcagaagacggcatacgagatcgcgcggtgtgactggagttcagacgtgtgctcttccgatctgatagtcagtgagcgaggaagc
17	MiSeq R4	caagcagaagacggcatacgagatcatgatcggtgactggagttcagacgtgtgctcttccgatctcgatagtcagtgagcgaggaagc
18	MiSeq R5	caagcagaagacggcatacgagatcgttaccagtgactggagttcagacgtgtgctcttccgatctcgatcagtcagtgagcgaggaagc
19	MiSeq R6	caagcagaagacggcatacgagattccttggtgtgactggagttcagacgtgtgctcttccgatcttagtcagtgagcgaggaagc
20	MiSeq R7	caagcagaagacggcatacgagataacgcattgtgactggagttcagacgtgtgctcttccgatctatagtcagtgagcgaggaagc
21	MiSeq R8	caagcagaagacggcatacgagatacaggtatgtgactggagttcagacgtgtgctcttccgatctgatagtcagtgagcgaggaagc
22	MiSeq R9	caagcagaagacggcatacgagataggtaagggtgactggagttcagacgtgtgctcttccgatctcgatagtcagtgagcgaggaagc
23	MiSeq R10	caagcagaagacggcatacgagataacaatgggtgactggagttcagacgtgtgctcttccgatctcgatcagtcagtgagcgaggaagc
24	MiSeq R11	caagcagaagacggcatacgagatactgtatcgtgactggagttcagacgtgtgctcttccgatcttagtcagtgagcgaggaagc
25	MiSeq R12	caagcagaagacggcatacgagataggtcgcagtgactggagttcagacgtgtgctcttccgatctatagtcagtgagcgaggaagc
26	pCAS9 GFP removal F	ggatccggagaaggaagagg
27	pCAS9 GFP removal R	ggatccgctgctgccgtt
28	pCAS9 mCh insertion F	gcaacggcagcagcggatcctccaagggcgaagaggac
29	pCAS9 mCh insertion R	cctcttccttctccggatcccttgtacagctcgtccattc
30	pET28a GRA29 25F	cagcggcctggtgccgcgcggcagccatatgagccgcttgaccgtgcgag
31	pET28a GRA29 R	ggtggtggtgctcgagtgcggccgcaagctttcaacgtgtccctcttccc
32	gRNA TGME49_288650 (GRA12) F	gggtatttcgatcgttgtgggttttagagctagaaatagc
33	gRNA TGME49_289150 F	ggacacgcagcctccgagaggttttagagctagaaatagc

Supplementary Table 1 continued

34	gRNA TGME49_313440 F	gcgacgggataaggtacgtggttttagagctagaaatagc
35	gRNA TGME49_254470 (MYR1) F	gggacaacacgcctctacgggttttagagctagaaatagc
36	gRNA TGME49_005250 ROP18 F	ggcgcacatgcaagaacaagttttagagctagaaatagc
37	gRNA TGME49_075470 Gra15 F	aagcgacttctaaacacgtggttttagagctagaaatagc
38	TGME49_288650 Homology mCH F	atttctactggataaggtgatatccgcaatctgctacacgtcgaaggctgtagtactgg
39	TGME49_288650 Homology mCH R	gagctgacgaaggcgttaagtaagatactcaaaggcgcgtgtcgactggaactacggtg
40	TGME49_289150 Homology mCH F	acgtgagtccagcggctttttccgctgtgctttttacggatcgaaggctgtagtactgg
41	TGME49_289150 Homology mCH R	gcttctctttcctggtcaccttcggtcattttttccacccgtcgactggaactacggtg
42	TGME49_313440 Homology mCH F	tttgtgtctgtgcagtgcgattcgcatttaattcgctggttcgaaggctgtagtactgg
43	TGME49_313440 Homology mCH R	gctagacgattagggaagcgttctgcgatcgcactcagatgtcgactggaactacggtg
44	TGME49_254470 Homology mCH F	atccgacgagtcactgaatccggaacaagaaatcagtggatcgaaggctgtagtactgg
45	TGME49_254470 Homology mCH R	aaagttgttcgggatttcccctggcacgttctcctgctttgtcgactggaactacggtg
46	TGME49_205250 Homology mCH F	atccagtcgttcactatttcctagcgcttgaggctgcccgattcgaaggctgtagtactg
47	TGME49_205250 Homology mCH R	acagcctgtcttgcggtgttaaatgtagcgcttgtcttcctgtcgactggaactacggtg
48	TGME49_275470 Homology mCH F	cccggtgcgccggcagtggtgcccatcttcgacgttgtctagtcgactggaactacggtg
49	TGME49_275470 Homology mCH R	agtcccccacggaagttttgaggacgttccggtggcgagggatcgaaggctgtagtactg
50	mCH Insert Analysis F	acgacttcaacgagatgttcc
51	mCH Insert Analysis R	gtgtcgaaacaagctgacac
52	TGME49_288650 Analysis F	cccggtttattcgactgcatg
53	TGME49_288650 Analysis R	acgcacgaaaacaaaacgg
54	TGME49_289150 Analysis F	gcacgttacttccagattcgtc
55	TGME49_289150 Analysis R	tctcttctctctgcaatgttcg
56	TGME49_313440 Analysis F	ccagttgtagctgccgtttc
57	TGME49_313440 Analysis R	gttccgaacgcttgaatgg
58	TGME49_254470 Analysis F	tgtagcggcgacggatg
59	TGME49_254470 Analysis R	aaactggactaggcgtctc
60	TGME49_275470 Analysis F	taagccgggtggtcattgtc
61	TGME49_275470 Analysis R	ccccctcgatcgtgtttgaa
62	TGME49_205250 Analysis F	gcttacggcgtctcatacca
63	TGME49_205250 Analysis R	aacgagatgttccgcgactt
