# Supplementary material for: A CRISPR platform for targeted in vivo screens identifies Toxoplasma gondii virulence factors in mice
Source: Nat Commun. 2019 Sep 3;10:3963. doi: 10.1038/s41467-019-11855-w (PMC6722137; doi:10.1038/s41467-019-11855-w)
Supplement: Supplementary file 2 — Reporting Summary [file 41467_2019_11855_MOESM2_ESM.pdf]

nature research

Corresponding author(s): Moritz Treeck

Last updated by author(s): 2019/05/26

Reporting Summary

Nature Research wishes to improve the reproducibility of the work that we publish. This form provides structure for consistency and transparency in reporting. For further information on Nature Research policies, see Authors & Referees and the Editorial Policy Checklist.

Statistics

For all statistical analyses, confirm that the following items are present in the figure legend, table legend, main text, or Methods section.

n/a	Confirmed	
☐	☒	The exact sample size (*n*) for each experimental group/condition, given as a discrete number and unit of measurement
☐	☒	A statement on whether measurements were taken from distinct samples or whether the same sample was measured repeatedly
☐	☒	The statistical test(s) used AND whether they are one- or two-sided<br>*Only common tests should be described solely by name; describe more complex techniques in the Methods section.*
☐	☒	A description of all covariates tested
☒	☐	A description of any assumptions or corrections, such as tests of normality and adjustment for multiple comparisons
☐	☒	A full description of the statistical parameters including central tendency (e.g. means) or other basic estimates (e.g. regression coefficient) AND variation (e.g. standard deviation) or associated estimates of uncertainty (e.g. confidence intervals)
☐	☒	For null hypothesis testing, the test statistic (e.g. *F*, *t*, *r*) with confidence intervals, effect sizes, degrees of freedom and *P* value noted<br>*Give P values as exact values whenever suitable.*
☒	☐	For Bayesian analysis, information on the choice of priors and Markov chain Monte Carlo settings
☒	☐	For hierarchical and complex designs, identification of the appropriate level for tests and full reporting of outcomes
☒	☐	Estimates of effect sizes (e.g. Cohen's *d*, Pearson's *r*), indicating how they were calculated

Our web collection on statistics for biologists contains articles on many of the points above.

Software and code

Policy information about availability of computer code

Data collection	Sequencing data was collected using HiSeq Control Software v3.4.0
Data analysis	Data was analyzed using Deseq2 v1.20.0, R v3.5.2, GraphPad PRISM v7, blast, fuzznuc from the EMBOSS suite (6.6.0), eCRISP cld-1.4.0 2017-01-19 as indicated in the methods sections.

For manuscripts utilizing custom algorithms or software that are central to the research but not yet described in published literature, software must be made available to editors/reviewers. We strongly encourage code deposition in a community repository (e.g. GitHub). See the Nature Research guidelines for submitting code & software for further information.

Data

Policy information about availability of data

All manuscripts must include a data availability statement. This statement should provide the following information, where applicable:

- Accession codes, unique identifiers, or web links for publicly available datasets
- A list of figures that have associated raw data
- A description of any restrictions on data availability

Raw sequencing counts are included in the manuscript.

Field-specific reporting

Please select the one below that is the best fit for your research. If you are not sure, read the appropriate sections before making your selection.

☒ Life sciences ☐ Behavioural & social sciences ☐ Ecological, evolutionary & environmental sciences

Life sciences study design

All studies must disclose on these points even when the disclosure is negative.

Sample size	For CRISPR screening we used the standard three technical replicates for the first experiment but subsequently used 5 in vitro and 8 in vivo replicates to improve confidence in the results. For in vivo virulence testing we used 5 mice per group so ensure robust data.
Data exclusions	One group of mice was excluded as infection dose was determined to be twice as anticipated. This precludes direct comparisons with WT parasite infections.
Replication	Parasite transfection experiments were replicated. Where an single observation is reported this is stated and consistent with replicated data in another parasite strain. For in vivo virulence tests, the number of experiments is clearly stated. Controls with previously published phenotypes were tested once. For one gene knockout (TGME49_289150) the inoculum for the repeat of the experiment was unusually high (double the other samples, see "Data exclusions").
Randomization	Randomization was used to reduce bias in mice selection and outcome assessment.
Blinding	For in vivo infections, the scientist carrying out the experiment was not aware of screening results and expected phenotypes. Microscopy slides where counted blinded such the the condition that was counted was not known to the person doing the data collection.

Reporting for specific materials, systems and methods

We require information from authors about some types of materials, experimental systems and methods used in many studies. Here, indicate whether each material, system or method listed is relevant to your study. If you are not sure if a list item applies to your research, read the appropriate section before selecting a response.

Materials & experimental systems

n/a	Involved in the study	
☐	☒	Antibodies
☐	☒	Eukaryotic cell lines
☒	☐	Palaeontology
☐	☒	Animals and other organisms
☒	☐	Human research participants
☒	☐	Clinical data

Methods

n/a	Involved in the study	
☒	☐	ChIP-seq
☒	☐	Flow cytometry
☒	☐	MRI-based neuroimaging

Antibodies

Antibodies used	Anti-Gra29 - generated by Covalab with recombinant protein.
Validation	Verified by testing against parasite knockout lines in western blot and IFA.

Eukaryotic cell lines

Policy information about cell lines

Cell line source(s)	Human foreskin fibroblasts where purchased by ATCC (ATCC® CCL-171™)
Authentication	no authentication was done.
Mycoplasma contamination	Cell lines were routinely tested for mycoplasma and were negative for this contamination.
Commonly misidentified lines (See ICLAC register)	*Name any commonly misidentified cell lines used in the study and provide a rationale for their use.*

Animals and other organisms

Policy information about studies involving animals; ARRIVE guidelines recommended for reporting animal research

Laboratory animals	C57BL/6 6-8 week old males from in house breeding.
Wild animals	The study did not include wild animals

Field-collected samples	The study did not include samples collected from the field.
Ethics oversight	All work was approved by the UK Home Office (project license PDE274B7D), the Francis Crick Institute Ethical Review Panel, and conforms to European Union directive 2010/63/EU.

Note that full information on the approval of the study protocol must also be provided in the manuscript.
