# Supplementary material for: A CRISPR platform for targeted in vivo screens identifies Toxoplasma gondii virulence factors in mice
Source: Nat Commun. 2019 Sep 3;10:3963. doi: 10.1038/s41467-019-11855-w (PMC6722137; doi:10.1038/s41467-019-11855-w)
Supplement: Supplementary file 3 — Description of Additional Supplementary Files [file 41467_2019_11855_MOESM3_ESM.pdf]

Description of Additional Supplementary Files

File Name: Supplementary Data 1
Description: **ME49 gRNA list.** All gRNAs designed against the ME49 genome and BLAST analysis hits against the GT1 genome; Related to Figure 1.

File Name: Supplementary Data 2
Description: **CRISPR_200_800_3200 libraries.** Raw and normalised 200, 800, 3200 gRNA sequencing counts, thresholded gene lfcs, and phenotype scores; Related to Figure 2

File Name: Supplementary Data 3
Description: **CRISPR_in vitro_in vivo.** Raw and normalized gRNAsequencing counts and lfcs, thresholded gene lfcs, phenotype and DISCO scores - in vivo experiment; Related to Figure 3
